# Light scattering of rectangular slot antennas: parallel magnetic vector vs perpendicular electric vector

**DOI:** 10.1038/srep18935

**Published:** 2016-01-07

**Authors:** Dukhyung Lee, Dai-Sik Kim

**Affiliations:** 1 Department of Physics and Astronomy and Center for Atom Scale Electromagnetism, Seoul National University, Seoul 08826, Republic of Korea

## Abstract

We study light scattering off rectangular slot nano antennas on a metal film varying incident polarization and incident angle, to examine which field vector of light is more important: electric vector perpendicular to, versus magnetic vector parallel to the long axis of the rectangle. While vector Babinet’s principle would prefer magnetic field along the long axis for optimizing slot antenna function, convention and intuition most often refer to the electric field perpendicular to it. Here, we demonstrate experimentally that in accordance with vector Babinet’s principle, the incident magnetic vector parallel to the long axis is the dominant component, with the perpendicular incident electric field making a small contribution of the factor of 1/|ε|, the reciprocal of the absolute value of the dielectric constant of the metal, owing to the non-perfectness of metals at optical frequencies.

Since the invention for microwave engineering^1^, rectangular slot antennas have shown its scalability even to visible range due to its simple design and has become a valuable component of plasmonics and near-field optics^2^,^3^,^4^,^5^,^6^,^7^,^8^,^9^. A prominent feature of high aspect ratio rectangular slot antennas is its striking polarization dependence: for normal incidence, only light which is consisted of the electric vector perpendicular to the long axis (therefore the magnetic vector parallel to the long axis) can pass through the antenna^10^,^11^,^12^. Following the convention referring the electric vector direction as polarization, this light is perpendicularly polarized to the long axis. Although the magnetic vector of light had been overlooked for a long time, due to negligible magnetic susceptibility at optical frequencies, in recent years, optical magnetism of various subwavelength systems is demonstrated^13^,^14^,^15^,^16^,^17^,^18^,^19^,^20^,^21^. Which is more important: electric field perpendicular to the long axis or the magnetic field parallel to it (Fig. 1(a))?

Contradicting the general convention regarding the preferred polarization, light scattering of a rectangular slot antenna in the perfectly conducting regime is actually determined by the incident magnetic vector, not the incident electric vector. We can easily figure out why, using vector Babinet’s principle. Vector Babinet’s principle states that if an infinitely thin screen of perfect electric conductor (PEC) and its complementary screen are illuminated by opposite polarizations (**E**_c_^(0)^ = *c***B**^(0)^, **B**_c_^(0)^ = −**E**^(0)^/*c*), then the scattered fields are related by **E** = *c***B**_c_ and **B** = −**E**_c_/*c* where *c* is the speed of light and the subscript c stands for complementary^22^. In other words, scatterings are the same except the exchanged roles of electric and magnetic fields. For the case of a thin linear antenna, which is the complementary screen of a rectangular slot antenna, the incident parallel electric vector accumulates charges at the far ends to generate scattered field, while the incident perpendicular magnetic vector cannot support excitation because the induced currents flowing on the front and back sides cancel each other (Fig. 1(b))^23^. According to vector Babinet’s principle, excitation of a rectangular slot antenna is determined by the incident parallel magnetic vector, irrespective of the incident perpendicular electric vector.

Vector Babinet’s principle has been extended to more realistic screens in many previous studies^24^,^25^. T. Zentgraf *et al*. experimentally demonstrated that the concept of Babinet’s principle can be applied to optical frequencies where metals are non-perfect. In that study, for split ring resonator and its Babinet complement, role is reversed between magnetic and electric fields even at optical frequencies^26^. Therefore, we can guess the behavior of a nano slot antennas at optical frequencies from the behavior of its Babinet complementary structure, a plasmonic nanowire antenna. Because a plasmonic nanowire antenna is driven by electric field at optical frequencies^27^, we expect that nano slot antenna will be driven by the magnetic field.

In this paper, we experimentally demonstrated that, even for a real metal screen of finite thickness and finite conductivity where the assumptions of Babinet’s principle fail, the incident parallel magnetic vector dominantly contributes to the light scattering while the incident perpendicular electric vector provides a minor contribution. To obtain respective contributions of the two incident vector components, we compared scatterings from two incident vector configurations similar to the ones shown in Fig. 1(a) using an extreme oblique incidence and normal-angle detection: at oblique incidence, the p-polarization has much weaker incident tangential electric vector giving the incident vector configuration similar with the left one in Fig. 1(a); oblique incidence of s-polarization has weaker magnetic one giving the incident vector configuration similar with the right one in Fig. 1(a)^28^,^29^.

## Experimental Setup

**Figure 2:**
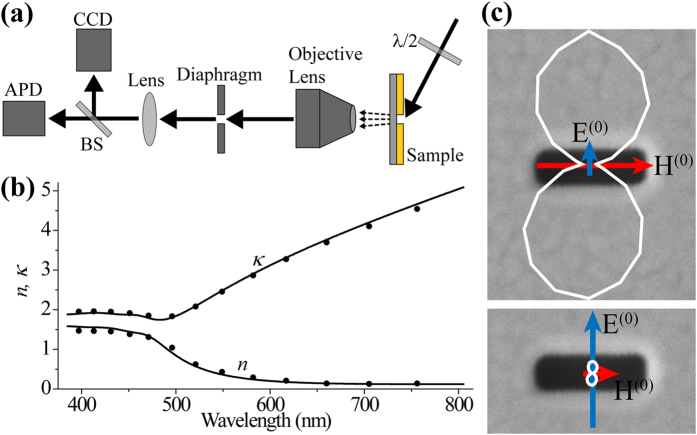
(**a**) Schematic diagram of the experiment. (**b**) Refractive index of the gold film obtained by spectroscopic ellipsometry. The lower and upper lines represent n and κ, real and imaginary parts, respectively. The circles are the tabulated data given by Johnson and Christy. (**c**) Illustrations of the incident tangential fields on SEM images of a 300 nm-long slot antenna for p-polarization (upper) and s-polarization (lower). By rotating the sample, we maintained the long axis to be parallel to the incident tangential magnetic field. The white lines are polar plots of polarization analyzed scattering intensity for a 300 nm-long slot antenna with the incident wavelength of 785 nm and the incident angle of 78°.

Figure 2(a) is a schematic diagram of the experiment. Rectangular slot antennas were illuminated by obliquely incident p- or s-polarized light. Scattering from a single slot antenna was collected by an objective lens (NA = 0.25) at the backside at normal direction and an avalanche photodiode (APD) detected the signal. A charge coupled device (CCD) camera was used to find the slot antenna. We can ignore the incident normal fields in this normal-direction detection, interpreting the scattering signal in terms of the incident tangential fields. Normal dipole induced by the normal incident fields hardly affects scattering signal in normal-direction detection because of its in-plane radiation characteristics^28^.

The rectangular slot antennas were perforated on a 100 nm thick gold film by focused ion beam with four different lengths (*l* = 100 nm, 200 nm, 300 nm, and 400 nm) and the same width (*w* = 100 nm). The gold film was deposited onto a sapphire substrate with a chromium adhesion layer by thermal evaporation. As discussed later in this paper, complex refractive index *ñ* of the metal is a key parameter to the relative contributions of the two vector components. We obtained the refractive index of the gold film as a function of wavelength λ by spectroscopic ellipsometry. Real and imaginary parts of the refractive index *ñ* ( = *n* + i*κ*) of the gold film are displayed as the lower and upper solid lines in Fig. 2 respectively; for reference, values given by Johnson and Christy^30^ are represented by filled circles.

Red and blue arrows in Fig. 2(c), respectively, illustrate the incident tangential magnetic and electric vectors for p-polarization (upper) and s-polarization (lower) on scanning electron microscope (SEM) images of a 300 nm-long slot antenna. In order to maintain the directions of the incident magnetic and electric vectors parallel and perpendicular to the long axis respectively, the sample was rotated by 90° when the polarization was changed from p to s. The relative strength between the two incident tangential vectors is determined by the incident angle.

We analyzed scattering polarization for a slot antenna (*l* = 300 nm) by placing a polarizer in front of the APD with the incident wavelength of 785 nm and the incident angle of 78°. White lines in Fig. 2(c) are polar plots of the polarization analyzed scattering intensities for the respective polarizations, showing the pre-eminence of p-polarization. Although both the incidences have proper vector direction for exciting the slot antenna, the scattering intensity for the p-polarization is much higher than the scattering intensity for the s-polarization. It shows that, in accordance with vector Babinet’s principle, the incident parallel magnetic vector is the dominant component in light scattering of the slot antenna still at λ = 785 nm.

## Results and Discussion

**Figure 3:**
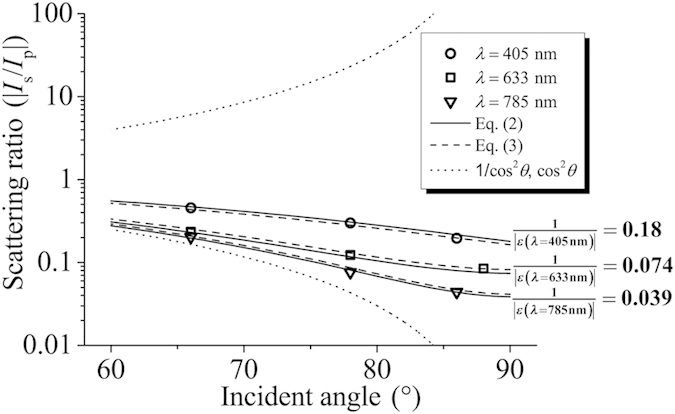
Scattering intensity ratio between s- and p-polarizations as a function of the incident angle *θ*. The circles, rectangles, and triangles are the experimental data for λ = 405 nm, 633 nm, and 785 nm respectively. *l* = 200 nm for λ = 633 nm and 785 nm, and *l* = 100 nm for λ = 405 nm. The solid lines are Eq. (2). Values of Eq. (2) at *θ* = 90°, which coincide with 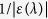, are displayed at the ends of the solid lines for the three wavelengths. The lower dotted line is cos^2^*θ* which is the intensity ratio of the incident tangential magnetic fields and the upper dotted line is 1/cos^2^*θ* which is the intensity ratio of the incident tangential electric fields. The dotted lines are Eq. (3).

To quantify the relative contributions of the incident parallel magnetic vector and incident perpendicular electric vector, we investigated scattering intensity ratio 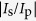. Figure 3 shows the scattering intensity ratio versus the incident angle *θ*. Circles, rectangles, and triangles in Fig. 3 are the experimental scattering intensity ratios for the λ = 405 nm, 633 nm, and 785 nm respectively. To keep the antenna length substantially sub-wavelength, for λ = 633 nm and 785 nm, we used the antenna of *l* = 200 nm; for λ = 405 nm, *l* = 100 nm was used.

The upper and lower dotted lines indicates 1/cos^2^*θ* and cos^2^*θ* which are the intensity ratios of the incident tangential electric fields and tangential magnetic fields, respectively. If the incident parallel magnetic field fully dominates scattering of the slot antenna, scattering intensity ratio should follow the lower dotted line and if the incident perpendicular electric field fully dominates, scattering intensity ratio should follow the upper dotted line. As shown in Fig. 3, measured scattering intensity ratios are closer to the lower dotted line than the upper one, confirming the dominance of the incident parallel magnetic vector. However, the scattering intensity ratio is not exactly same with the intensity ratio of the incident parallel magnetic fields. The deviation from cos^2^*θ* indicates that there is some contribution from the incident perpendicular electric field.

We found that the contribution of the incident perpendicular electric vector is approximately 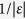 times smaller than the contribution of the incident parallel magnetic vector, where *ε* is the relative permittivity of the metal. The factor 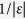 clearly appears at grazing incidences where the incident tangential fields are almost solely magnetic for p-polarization and solely electric for s-polarization. The values of 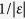 for λ = 405 nm, 633 nm, and 785 nm are 0.18, 0.074, and 0.039 respectively, which are very close to the experimental data, 0.20, 0.085, and 0.044, at grazing incidences (*θ* = 86° or 88°).

Considering the mechanism of the slot antenna scattering reveals where the factor 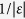 comes from. Distortion of the surface current in the vicinity of the slot is the source of the light scattering. The surface current distortion is proportional to the surface current flowing on the flat surface. Because the surface current on the flat surface is proportional to the total tangential magnetic field, the scattering amplitude is also proportional to the total tangential magnetic field on the flat surface. The total tangential magnetic field, a superposition of the tangential components of the incident and reflected magnetic fields, can be obtained using Fresnel coefficients^31^. Accordingly, the scattering intensity ratio is expressed as follows:


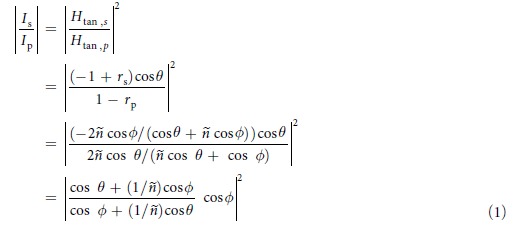


where *r*_s_ and *r*_p_ are Fresnel reflection coefficients and 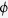 is the complex refraction angle^32^. Even in visible range, refractive index *ñ* of a metal is usually large enough to approximate 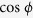 as 1. Thus, we modified Eq. (1) in a more intuitive form as follows:


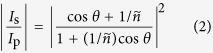


Equation (2) displayed as solid lines in Fig. 3 shows good agreement with experimental data at all incident angles. Equation (2) goes to 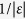 as the incident angle goes to 90°. From the positions of cos*θ*, we can take 1/*ñ* as the contribution of the incident perpendicular electric field to the scattering amplitude. To put it briefly, at a given incident angle, the incident electric field induces 1/*ñ* times weaker surface current and in turn scattering amplitude, than the incident magnetic field does. In terms of the scattering intensity, contribution of the incident electric field is smaller by a factor of 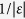 (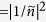).

**Figure 4:**
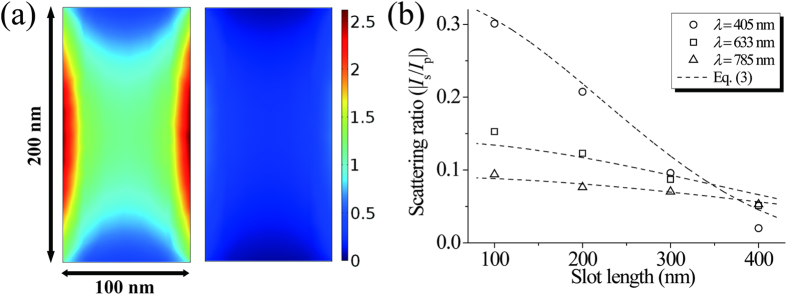
(**a**) Simulated electric field amplitude profile at the exit side of a freestanding gold aperture of *l* = 200 nm and *w* = 100 nm for p-polarization (left) and s-polarization (right) at λ = 785 nm and *θ* = 85°. (**b**) Scattering intensity ratio between s- and p-polarizations as a function of the slot length at the incident angle of 78°. The circles, rectangles, and triangles are the experimental data for λ = 405 nm, 633 nm, and 785 nm respectively. The dashed lines are Eq. (3).

We further investigated on the rectangular shape effect. Figure 4(a) shows the simulated electric field amplitude profile at the exit side of a freestanding gold aperture of *l* = 200 nm and *w* = 100 nm for p-polarization (left) and s-polarization (right) at λ = 785 nm and *θ* = 85°. Simulation was conducted with COMSOL Multiphysics 5.1. As indicated by the color scale, the excited field amplitude is 4~5 times higher for p-polarization. It supports our above argument because the 4~5 times difference is consistent with the refractive index of the gold, *ñ* = 0.12 + 4.92i, at λ = 785 nm. An important point is that for our experimental parameters, both the p- and s-polarization are dominated by the same fundamental mode. Therefore, the difference in s- and p- field polarizations does not originate from different mode excitation, but depends mostly on the refractive index of the metal.

Figure 4(b) shows the scattering intensity ratio versus the slot length for *θ* = 78°. Decrease of the scattering intensity ratio with increasing the slot length can be attributed to the phase retardation of the incident fields over the antenna. In the experimental geometry, the phase of the incident field varies in the width direction for the p-polarization and in the length direction for the s-polarization. Because increasing the slot length makes the slot antenna feel more phase retardation for the s-polarization, the scattering intensity ratio decreases. By assuming the fundamental mode of the slot antenna to be the same one in PEC condition^33^, Eq. (2) is modified to include the phase retardation factor as follows:


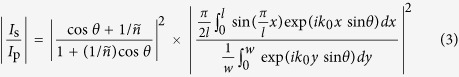


where *k*_0_ is the free space wavevector. Equation (3) is displayed as dashed lines in Figs 3 and 4(b). As shown in the good agreement of the experimental data and Eq. (3), the phase retardation effect explains the further decreasing scattering intensity ratio with increasing slot length. Deviation of the experimental value from Eq. (3) for *l* = 300 and 400 nm at λ = 400 nm in Fig. 4(b) may possibly be due to the higher mode excitation and resonance effect. If the size of the slot antenna is sufficiently smaller than the wavelength, the phase retardation effect vanishes and Eq. (3) is reduced to Eq. (2) as shown in Fig. 3 where the solid lines and the dashed lines are nearly the same.

## Conclusion

We demonstrated that light scattering of rectangular slot antennas is mainly dependent on the incident parallel magnetic vector. The contribution of the incident perpendicular electric field is smaller by a factor of 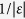 because the incident electric field induces 1/*ñ* times weaker surface current at the metal surface than the incident magnetic field does. For PEC, the factor 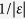 vanishes and light scattering of a slot antenna is fully dominated by the incident parallel magnetic field as expected from vector Babinet’s principle. The degree of dominance of the incident magnetic field on the slot antenna scattering is determined by the permittivity of the metal, not by the specific geometry of the slot. Because the factor 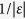 provides useful and intuitive estimation of the relative contributions of the incident magnetic and electric fields, we expect that our finding is beneficial for designing optical magnetism and metamaterials.

## Additional Information

**How to cite this article**: Lee, D. and Kim, D.-S. Light scattering of rectangular slot antennas: parallel magnetic vector vs perpendicular electric vector. *Sci. Rep*. **6**, 18935; doi: 10.1038/srep18935 (2016).

## Figures and Tables

**Figure 1:**
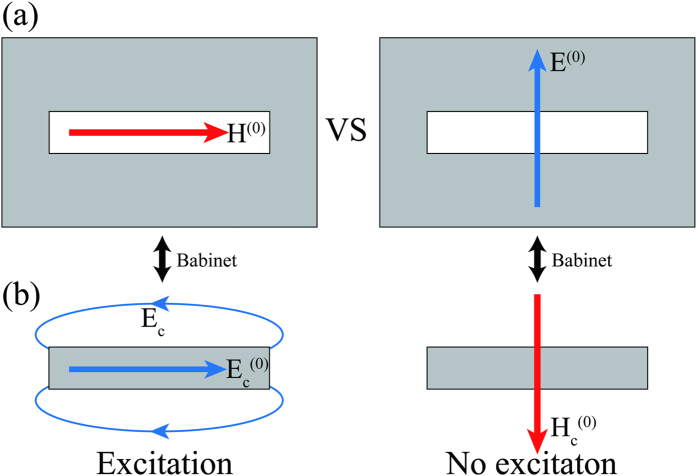
(**a**) For a normal incidence, the proper incident polarization for rectangular slot antenna functioning is consisted of the incident electric vector perpendicular to the long axis (right) and the incident magnetic vector parallel to it (left). (**b**) A thin linear antenna is excited by the incident parallel electric vector (left), while cannot be excited by the incident perpendicular magnetic vector (right). Because a thin linear antenna is the Babinet complementary structure of a rectangular slot antenna, roles of the magnetic and electric fields are reversed.
